# Membrane lipid unsaturation as physiological adaptation to animal longevity

**DOI:** 10.3389/fphys.2013.00372

**Published:** 2013-12-17

**Authors:** Alba Naudí, Mariona Jové, Victòria Ayala, Manuel Portero-Otín, Gustavo Barja, Reinald Pamplona

**Affiliations:** ^1^Department of Experimental Medicine, University of Lleida-Biomedical Research Institute of Lleida (UdL-IRBLleida)Lleida, Spain; ^2^Department of Animal Physiology II, Complutense UniversityMadrid, Spain

**Keywords:** fatty acid biosynthesis, membrane unsaturation, oxidative damage, peroxidizability index, phylogenomic analysis, rate of aging, reactive carbonyl species

## Abstract

The appearance of oxygen in the terrestrial atmosphere represented an important selective pressure for ancestral living organisms and contributed toward setting up the pace of evolutionary changes in structural and functional systems. The evolution of using oxygen for efficient energy production served as a driving force for the evolution of complex organisms. The redox reactions associated with its use were, however, responsible for the production of reactive species (derived from oxygen and lipids) with damaging effects due to oxidative chemical modifications of essential cellular components. Consequently, aerobic life required the emergence and selection of antioxidant defense systems. As a result, a high diversity in molecular and structural antioxidant defenses evolved. In the following paragraphs, we analyze the adaptation of biological membranes as a dynamic structural defense against reactive species evolved by animals. In particular, our goal is to describe the physiological mechanisms underlying the structural adaptation of cellular membranes to oxidative stress and to explain the meaning of this adaptive mechanism, and to review the state of the art about the link between membrane composition and longevity of animal species.

## Introduction

**Figure 1:**
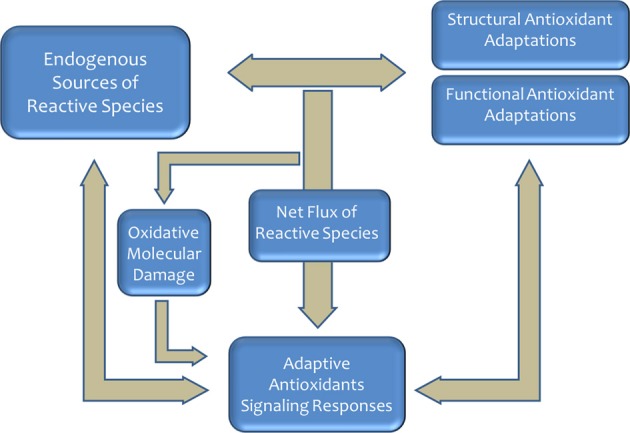
**Aerobic life produces reactive species that required the emergence and selection of antioxidant defense systems**. Redox state is an important selective pressure faced by most organisms, and a myriad of mechanisms have evolved to regulate and adjust this process. In homeostatic conditions, the sytem play a key role in the aging process and the determination of the longevity.

The appearance of oxygen in the terrestrial atmosphere represented an important selective pressure for ancestral living organisms and contributed toward setting up the pace of evolutionary changes in structural and functional systems (McCord, 2000; Lane, 2002; Embley and Martin, 2006; Pamplona and Costantini, 2011). The evolution of using oxygen for efficient energy production served as a driving force for the evolution of complex organisms (Lane, 2002; Schirrmeister et al., 2013). The redox reactions associated with its use were, however, responsible for the production of reactive species [reactive oxygen species (ROS) and reactive carbonyl species (RCS)] with damaging effects due, basically, to oxidative chemical modifications of essential cellular components (Halliwell and Gutteridge, 2007). Consequently, aerobic life required the emergence and selection of antioxidant defense systems (Halliwell, 1999). As a result, a high diversity in molecular and structural antioxidant defenses evolved (Pamplona and Costantini, 2011). Nevertheless, the balance between oxidant production systems and antioxidant defenses is adjusted in a species-specific way to generate a net flux of oxidant (see Figure 1) for maintaining antioxidant responses through redox signaling pathways perfectly integrated in the cellular metabolic machinery. This oxidative stress has become a universal constraint of life-history evolution in animals and a modulator of phenotypic development (Dowling and Simmons, 2009; Pamplona and Costantini, 2011).

Living organisms on the Earth which are divided into three major domains—Archaea, Bacteria, and Eucarya—, probably came from a common ancestral cell. The cell membrane is a key dynamic structural component of a cell, and lipid molecules are essential for cell membranes (Itoh et al., 2001; Lombard et al., 2012). All cell membranes are composed of glycerol phosphate phospholipids, and this commonality argues for the presence of such phospholipids in the last common ancestor (Lombard et al., 2012). Therefore, all living organisms have lipid membranes.

Biological membranes are dynamic structures that generally consist of bilayers of amphipathic molecules held together by non-covalent bonds (Yeagle, 1993; Vance and Vance, 1996). Phospholipids, the predominant membrane lipids in eukaryotic cells, are made up of a glycerol backbone with a hydrophilic headgroup bound to carbon 3, and fatty acids to C1 and C2. Phospholipids are a large group of diverse molecules that participate in a wide range of biological processes (Dowhan, 1997). This diversity requires complex metabolic and regulatory pathways (Yeagle, 1993; Vance and Vance, 1996). Indeed eukaryotic cells, to monitor cell membrane composition and to adjust lipid synthesis accordingly (Dobrosotskaya et al., 2002), invest around 5% of their genes to synthesize all of these lipids (Van Meer et al., 2008).

**Figure 2:**
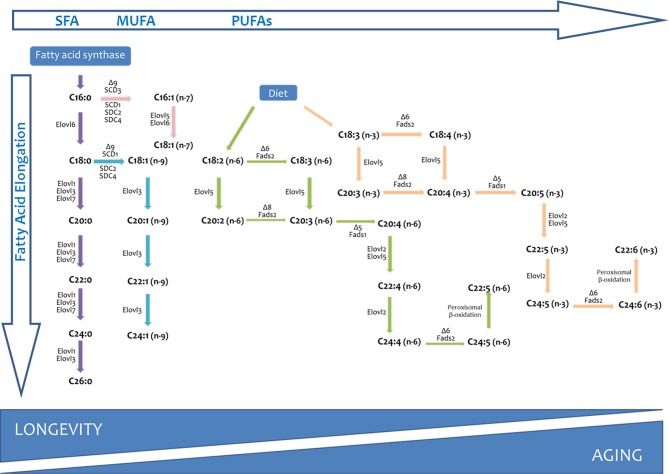
**Long chain and very long-chain fatty acid biosynthesis in vertebrates**. The long chain saturated fatty acids and unsaturated fatty acids of the *n*-7 and *n*-9 series can be synthesized from palmitic acid (C16:0) produced by the fatty acid synthase (FAS). Long-chain fatty acids of the *n*-6 and *n*-3 series can only be synthesized from precursors obtained from dietary precursors (DIET). *Elovl*, elongation of very long chain fatty acids (fatty acid elongase); Fads, fatty acid desaturase.

The phospholipid acyl chains are saturated (SFA), monounsaturated (MUFA), or polyunsaturated (PUFA) hydrocarbon chains that normally vary from 14 to 22 carbons in length (Wallis et al., 2002). In eukaryotic cells from vertebrate species, for example, the average chain length of a biological membrane is strictly maintained around 18 carbon atoms, and the relative distribution between SFAs and UFAs follows a ratio of 40:60 (Pamplona, 2008). Substrate precursors for UFA biosynthesis are generally SFA that are products of fatty acid synthase (FAS), as well as essential fatty acids from dietary sources. The desaturase and elongase enzymes, which are conserved across kingdoms, as well as the peroxisomal beta-oxidation pathway, will allow cells to obtain all the diversity of fatty acids present in a cellular membrane (Nakamura and Nara, 2004; Guillou et al., 2010) (see Figure 2). Finally, the deacylation-reacylation cycle will be the mechanism responsible for the particular fatty acid composition of cell membranes.

In the following paragraphs, we analyze the adaptation of biological membranes as dynamic structural defense against reactive species evolved by animals. In particular, our goal is to describe the physiological mechanisms underlying the structural adaptation of cellular membranes to oxidative stress and to explain the meaning of this adaptive mechanism, and to review the state of the art about the link between membrane composition and longevity of animal species.

## Lipid peroxidation of biological membranes: signaling vs. citotoxicity

The physico-chemical properties of the membrane bilayer and the chemical reactivity of the fatty acids that compose the membrane are two inherent traits of the membrane phospholipids that determine their susceptibility to oxidative damage (Pamplona et al., 2002a,b,c; Hulbert et al., 2007; Pamplona, 2008). The first property is related to the fact that oxygen and reactive species are more soluble in the fluid lipid bilayer than in the aqueous solution (Moller et al., 2005; Gamliel et al., 2008). Consequently, membrane lipids become primary targets of oxidative damage. The second and more significant property is related to the fact that PUFA residues of phospholipids are extremely sensitive to oxidation, their sensitivity increasing exponentially as a function of the number of double bonds per fatty acid molecule (Holman, 1954; Bielski et al., 1983). Consequently, PUFA side chains (with two or more double bonds) are much more easily attacked by radicals than are SFA (no double bonds) or MUFA (one double bond) side chains. In this scenario, from a given membrane fatty acid profile it is possible to calculate its peroxidizability index (PI) by combining this composition with the relative susceptibility of individual fatty acids to peroxidation. So, PI is an approach to the relative susceptibility of a given membrane fatty acid composition to peroxidative damage. The higher the number the more susceptible, the lower the value of PI, the more resistant to lipid peroxidation is the membrane bilayer (Pamplona et al., 2002a,b,c; Hulbert et al., 2007).

Lipid peroxidation generates hydroperoxides as well as endoperoxides, which undergo fragmentation to produce a broad range of reactive intermediates called RCS with three to nine carbons in length, the most reactive being α,β-unsaturated aldehydes [4-hydroxy-trans-2-nonenal (HNE) and acrolein], di-aldehydes [malondialdehyde (MDA) and glyoxal], and keto-aldehydes [4-oxo-trans-2-nonenal (ONE) and isoketals] (Esterbauer et al., 1991; Catalá, 2009; Zimniak, 2011; Fritz and Petersen, 2013). 2-Hydroxyheptanal and 4-hydroxyhexenal are other significant aldehydic products of lipid peroxidation of PUFAs. These carbonyl compounds, ubiquitously generated in biological systems, have unique properties contrasted with other reactive species. For instance, compared with ROS, reactive aldehydes have a much greater half-life (i.e., minutes to hours instead of nanoseconds to microseconds for most ROS). Further, the non-charged structure of RCS allows them to migrate easily through hydrophobic membranes and hydrophilic cytosolic media, thereby extending the migration distance far from the generation site (Pamplona, 2008).

These compounds have specific physiological signaling roles inducing adaptive responses driven to decrease oxidative damage and improve antioxidant defenses (Pamplona, 2008, 2011; Higdon et al., 2012). Two of these mechanisms involved in the prevention of oxidative damage effects are: (i) the regulation of uncoupling protein activity (Echtay et al., 2003; Brand et al., 2004), and (ii) the activation of the antioxidant response signaling pathway that includes the expression of enzymes such as glutathione-S-transferase (GST) specifically designed to detoxify reactive carbonyl compounds (Wakabayashi et al., 2004; Copple et al., 2008; Giles, 2009; Maher and Yamamoto, 2010). As important as GST is for these adaptive mechanisms, is the role GP × 4 (phospholipid hydroperoxide glutathione peroxidase) has in restoring reduced state of membrane fatty acids from phospholipids to ensure membrane lipid homeostasis (Imai and Nakagawa, 2003; Brigelius-Flohé, 2006; Conrad et al., 2007). GP × 4 gene structure, expression and activity is likely to have evolved in a coherent fashion to cope with prooxidant conditions.

Based on the features mentioned above these carbonyl compounds can be, however, more destructive than ROS and may have far-reaching damaging effects on target sites within or outside membranes. Carbonyl compounds react with nucleophilic groups in macromolecules (lipoxidation reactions) like proteins (Thorpe and Baynes, 2003), DNA (West and Marnett, 2006), and aminophospholipids (Naudi et al., 2013), among others, resulting in their chemical, nonenzymatic, and irreversible modification and formation of a variety of adducts and crosslinks collectively named Advanced Lipoxidation Endproducts (ALEs) (Thorpe and Baynes, 2003; Pamplona, 2011).

Consequently, the high concentration of PUFAs in cellular membrane phospholipids not only makes them prime targets for reaction with oxidizing agents but also enables them to participate in long free radical chain reactions. The regulatory function and cytotoxicity of the lipid peroxidation-derived aldehydes hinges on its abundance, reactivity, and half-life. It is plausible to postulate that a low degree of fatty acid unsaturation in cellular membranes could be advantageous by decreasing their sensitivity to lipid peroxidation. This would also protect other molecules against lipoxidation-derived damage. In other words, it is proposed that membrane unsaturation acts as a structural adaptive system and it is related to the animal longevity.

## Membrane unsaturation and animal longevity

Findings from different experimental paradigms recently link membrane unsaturation and lipoxidation-derived molecular damage to longevity: (i) changes that occur in individuals during aging; (ii) different maximum longevity that are characteristic of species and the different longevity of strains and specific mutants within species; and (iii) physiological treatments that alter rate of aging and thus longevity.

### Is aging due to increase of membrane unsaturation and lipoxidation reactions?

It has now been documented that there is an age-associated increase in membrane PI and lipoxidation-derived molecular damage (see Table 1). In general, PI increases during aging in an organ-dependent way. This is mainly due to decreases in the less unsaturated linoleic (LA, 18:2*n*-6) and linolenic (LNA, 18:3*n*-3) acids and to increases in the highly unsaturated arachidonic acid (AA, 20:4*n*-6), and docosotetra- penta- and –hexaenoic [22:4*n*-3, 22:5*n*-3, and 22:6*n*-3 (DHA), respectively] acids. In a similar way, the lipoxidative damage, which has been measured in tissue homogenates and mitochondria, also increases with age. Tissues that are composed of long-lived, postmitotic cells, such as the brain, heart, and skeletal muscle, tend to accrue relatively greater amounts of damage than those composed of short-lived non-mitotic cells. In this line, lipofuscin, a complex age-pigment derived from lipoxidation reactions considered a hallmark of aging, also shows an accumulation that correlates with age (Tsuchida et al., 1987; Terman and Brunk, 2004).

**Table 1:** **Effect of aging on membrane peroxidizability index and lipoxidation-derived molecular damage in tissues from different species**.

**Tissue**	**Species**	**Age(s)**	**Change with aging**	**Change with aging in**	**References**
			**in Peroxidizability**	**Lipoxidation-derived**	
			**Index (PI)**	**molecular damage**	
Whole	*Drosophila*	From 10 to 50 days	Increase	Increase	Magwere et al., 2006
Whole	*Drosophila*	From 5 to 40 days	Increase	Increase	Jacobson et al., 2010
Brain	Mouse	6 vs. 24 months	Increase	Increase	Arranz et al., 2013
Spleen	Mouse	6 vs. 24 months	Increase	Increase	Arranz et al., 2013
Heart	Rat	8 vs. 30 months	Increase	Increase	Ayala et al., 2006
Liver	Rat	8 vs. 30 months	Increase	Increase	Ayala et al., 2006
Liver mitochondria	Rat	6, 18, 28 months	Increase	Increase	Lambert et al., 2004
Liver microsomes and mitochondria	Rat	6, 12, 24 months	Increase	n.d.	Laganiere and Yu, 1993
Erythrocyte membranes	Human	From 20 to 90 years	Increase	n.d.	Rabini et al., 2002

n.d., not determined.

The singular importance of membrane unsaturation in the aging process is highlighted by studies showing age-related changes in membrane physico-chemical properties. During aging, the membrane fatty acid profile changes with increased peroxidizable PUFAs (see Table 1). PUFAs have lower melting points than SFAs, and consequently, a relative increase in the PUFA content of a membrane would be expected to render the membrane more fluid. Based solely on the fatty acid composition, membranes from older animals should exhibit greater fluidity than those from younger individuals. Paradoxically, the opposite is systematicaly reported; membrane fluidity decreases with age (reviewed in Hulbert et al., 2007). The reasons for the changes of membrane fluidity can be explained in two ways: (1) a change caused by fatty acid chain composition and (2) a change in the cholesterol content of the membrane. The paradox described above is explained by considering that cellular components undergo increased oxidative damage with time. Because PUFAs are more vulnerable to oxidative attack, they experience greater lipid oxidative damage, and the resulting RCSs have been shown to contribute significantly to membrane rigidity and loss of its function (Laganiere and Yu, 1987, 1993; Yu et al., 1992; Yu, 2005; Naudi et al., 2013). A special mention to the recent indications that membrane microdomains (i.e., rafts) also undergo an aging process not necessarily identical to that in the bulk membrane (given the differential biochemical composition between raft and non-raft domains) (Tomoiu et al., 2007; Ohno-Iwashita et al., 2010; Fabelo et al., 2012). These observations apparently correlate with increased membrane microviscosity that parallels changes in both PUFA and cholesterol (which would have opposed effects), and very interestingly, they appear to be accelerated by neurodegenerative processes.

Studies on possible causes of age-associated increase in membrane unsaturation (see Table 1) indicated that activities of some of the desaturase and elongase enzymes that participate in the PUFA biosynthesis pathway, as well as the peroxisomal beta-oxidation pathway increase with age suggesting the presence of a generalizable pattern. The reasons (dysfunctionality or adaptation?) for the increase in these enzymatic activities during aging remain unknown. In any case, this pattern favors a high age-associated membrane unsaturation that, in turn, is highly susceptible to lipid peroxidation (independently of the presence or absence of an increase in age-associated ROS production, or defects in molecular removal or repair) and to the induction of a high steady-state level of lipoxidation derived molecular damage. Therefore, the available data tend to favor the view that the increase in membrane unsaturation is a key factor responsible for age-related accrual of molecular lipoxidative damage.

### Are interspecies variations in longevity related to corresponding differences in membrane unsaturation and lipoxidation-derived molecular damage?

The first indication of a connection between membrane fatty acid composition and maximum longevity was the report by Pamplona et al. (1996) which demonstrated that the PI of liver mitochondria from rats, pigeons and humans was strongly correlated with their respective longevity. Later, it was shown that this was the case for a wide range of tissues and animal species including mammals, birds, insects and crustaceans. Unfortunately, no data are currenly available for reptiles, amphibians, fishes, and many invertebrates.

Thus, it has been found that long-lived animals (birds and mammals, including humans) have a lower degree of total tissue and mitochondrial fatty acid unsaturation and PI than short-lived ones (Table 2). In agreement with this, it was demonstrated that in long-lived animal species a low degree of total tissue and mitochondrial fatty acid unsaturation was accompanied by a low sensitivity to *in vivo* and *in vitro* lipid peroxidation and a low steady-state level of lipoxidation-derived adducts in both tissue and mitochondrial proteins from organs like skeletal muscle, heart, liver, and brain (Pamplona et al., 2002a,b,c; Pamplona, 2008; Pamplona and Barja, 2011). Reinforcing this idea of low lipoxidative damage in long-lived species, lipofuscin also showed an accumulation rate that inversely correlates with longevity (Terman and Brunk, 2004). These findings were consistent with the negative correlation previously observed between longevity and the sensitivity to lipid autoxidation of mammalian kidney and brain homogenates (Cutler, 1985).

**Table 2:** **Comparative studies between membrane unsaturation (peroxidizability index, PI) and longevity in animal species (by chronological order)**.

**Species compared**	**Organ**	**PI long-lived**	**References**
		**species**	
Rat-Pigeon-Human	Liver mitochondria	Lower	Pamplona et al., 1996
SAM-R/1 vs. SAM-P/1 mice	Liver	Lower	Park et al., 1996
8 mammalian species	Liver mitochondria	Lower	Pamplona et al., 1998
Rat vs. pigeon	Heart mitochondria	Lower	Pamplona et al., 1999a,b
Mouse vs. canary	Heart	Lower	Pamplona et al., 1999a,b
Mouse vs. parakeet	Heart	Lower	Pamplona et al., 1999a,b
Rat vs. pigeon	Liver mitochondria	Lower	Gutiérrez et al., 2000
Rat vs. pigeon	Heart mitochondria and microsomes	Lower	Gutiérrez et al., 2000
8 mammalian species	Heart	Lower	Pamplona et al., 2000a,b
7 mammalian species	Liver	Lower	Pamplona et al., 2000a,b
8 mammalian species	Liver mitochondria	Lower	Herrero et al., 2001
Rat vs. pigeon	Skeletal muscle	Lower	Portero-Otín et al., 2004
Mouse, parakeet, canary	Brain	Lower	Pamplona et al., 2005
8 mammalian species	Heart	Lower	Ruiz et al., 2005
Strains of mice (Idaho, Majuro, and WT)	Skeletal muscle and liver	Lower	Hulbert et al., 2006a,b
Naked-mole rat vs. mouse	Skeletal muscle mitochondria and Liver mitochondria	Lower	Hulbert et al., 2006a,b
12 mammalian species and 9 bird species	Skeletal muscle	Lower	Hulbert et al., 2007
10 mammalian species and 8 bird species	Liver mitochondria	Lower	Hulbert et al., 2007
Queen honey bees vs. workers	Head, thorax, abdomen	Lower	Haddad et al., 2007
42 mammalian species	Skeletal muscle	Lower^*^	Valencak and Ruf, 2007
13 bird species	Heart	Lower	Buttemer et al., 2008
Echidna vs. mammals	Liver, liver mitochondria, and Skeletal muscle	Lower	Hulbert et al., 2008
Humans (offspring of nonagenarians vs. control)	Erythrocytes	Lower	Puca et al., 2008
*D. melanogaster* (long-lived mutant strains)	Whole organism and mitochondria	Lower	Sanz et al., 2010
*C. elegans* (long-lived mutant strains)	Whole organism	Lower	Shmookler Reis et al., 2011
Rat vs. pigeon	Erythrocytes, heart, kidney, liver, skeletal muscle (whole tissue and mitochondria)	Lower	Montgomery et al., 2011
5 marine molluscs species	Whole mitochondria	Lower	Munro and Blier, 2012
Exceptionally-old mice	Brain, spleen	Lower	Arranz et al., 2013
Humans (Middle aged offspring of nonagenarians vs. control group)	Plasma	Lower	Gonzalez-Covarrubias et al., 2013
Long-lived vs. short-live mouse (*P. leucopus* vs. *M. musculus*)	Skeletal muscle mitochondria	Lower	Shi et al., 2013
Wild-type vs. long-lived Ames dwarf mice	Skeletal muscle, heart, liver, mtLiver, brain	Lower^**^	Valencak and Ruf, 2013
11 mammalian species	Plasma	Lower	Jové et al., 2013a,b
*D. melanogaster* (wild-type strains)	Whole organism and mitochondria	Lower	Naudi et al., 2013

* Results obtained after correction for body weight and phylogeny showed that longevity decreases as the ratio of n-3 to n-6 PUFAs increases. No relation between longevity and PI was found;

** No significant differences were observed for brain.

All these observations made at cell-tissue level can be interestingly extended to plasma lipids. Thus, in a recent study (Jové et al., 2013a,b) the plasma lipidomic profile by using high-throughput lipidome profiling technologies of 11 mammalian species ranging in maximum longevity from 3.5 to 120 years was determined. Using a non-targeted approach about 14,000 lipid species in plasma was detected, and the multivariate analyses separated perfectly 11 groups, indicating a specific signature for each animal species which accurately predicts animal longevity. Regression analysis between lipid species and longevity revealed that long-chain free fatty acid concentrations, PI, and lipid peroxidation-derived products correlated in a specific and significant way. Thus, the greater the longevity of a species, the lower is its plasma long-chain free fatty acid concentration, peroxidizability, and lipid peroxidation-derived products content, suggesting that the lipidomic signature is an optimized feature associated with animal longevity.

While longevity can differ dramatically between mammal and bird species, there can also be significant longevity differences within a species. Thus, populations of two wild-derived strains of mice display extended longevity (both mean and maximum longevity) compared to genetically heterogenous laboratory mice when kept under identical conditions (Miller et al., 2002). The PI of both skeletal muscle and liver phospholipids of the two wild-type mice strains with the extended longevity was significantly smaller than that of the laboratory mice (Hulbert et al., 2006a,b). This is notable because, since the different mice strains were fed the identical diet, it shows that the differences in membrane fatty acid composition between species are not determined by dietary differences but is genetically regulated. In a similar way, in the senescence-accelerated mouse (SAM) strain, the SAM-prone mice had greater levels of the highly polyunsaturated peroxidation-prone fatty acids 22:6 *n*-3 and 20:4 *n*-6 and lower levels of the more peroxidation-resistant 18:2 *n*-6 PUFA in their membranes, and consequently had a greater PI than the SAM-resistant mice (Park et al., 1996). SAM-prone mice also showed greater degrees of lipid peroxides in their tissues than do SAM-resistant mice.

In this context, it is also of great interest to know and discern the degree of membrane unsaturation and the steady-state levels of lipoxidative damage in physiological systems from exceptionally long-lived specimens. Thus, in a recent work (Arranz et al., 2013), adult (28 weeks), old (76 weeks), and exceptionally old (128 weeks) BALB/c female mice were used. Brain and spleen were analyzed for membrane fatty acid composition and markers of lipoxidative molecular damage. The results showed significantly lower PI and lipoxidation-derived protein damage in brain and spleen from exceptionally old animals when compared to old specimens, and in a range analogous to adult animals. Therefore, low susceptibility to lipid peroxidation and maintenance of adult-like molecular lipoxidative damage could be key factors for longevity achievement.

Comparing biological processes in closely-related species with divergent longevity can also be a powerful approach to study mechanisms of longevity. Thus, the skeletal muscle mitochondria from long-lived white-footed mouse *Peromyscus leucopus* (MLSP = 8 years) display lower levels of isoprostanes (lipid peroxidation-derived compounds) than the common laboratory mouse, *Mus musculus* (MLSP = 3.5 years) (Shi et al., 2013).

Two exceptionally long-living mammalian species (naked mole-rats and echidnas) also have membrane fatty acid profiles that are resistant to lipid peroxidation as one would predict from their longevities. Thus, when membrane fatty acid composition was measured in tissues from naked mole-rats, the longest-living rodents known with a recorded longevity exceeding 28 years (Buffenstein, 2005), it was found that they have very low levels of 22:6*n*-3 in their tissue phospholipids compared to mice. Although both mice and naked mole-rats have similar levels of total UFAs in their tissue phospholipids, the low 22:6*n*-3 levels of the naked mole-rats result in lower PI and more peroxidation-resistant membranes in skeletal muscle and liver mitochondria (Hulbert et al., 2006a,b; Mitchell et al., 2007). In a similar way, the echidna *Tachyglossus aculeatus*, a monotreme mammal from Australia that is exceptionally long-living with a documented longevity of 50 years, also had a membrane composition resistant to lipid peroxidation (Hulbert et al., 2008). Accordingly, membrane lipids of echidna tissues (skeletal muscle, liver, and liver mitochondria) were found to have a lower content of PUFAs and a higher content of MUFAs, resulting in a low PI and, consequently, indicating that the cellular membranes of echidnas is peroxidation-resistant (Hulbert et al., 2008).

Honeybees (*Apis mellifera*) and flyes (*D. melanogaster*) provide another example of variation in longevity within a species that extend previous findings in vertebrates to invertebrates. In the honey bee, depending on what they are fed, female eggs become either workers or queens (Winston, 1987). Hence, queens and workers share a common genome. However, the longevity of queens is an order-of-magnitude greater than that of workers. In order to test if differences in membrane composition could be involved the fatty acid composition of phospholipids of queen and worker honey bees were compared (Haddad et al., 2007). The cell membranes of both young and old honey bee queens were highly monounsaturated with very low content of PUFAs. Newly emerged workers show a similar membrane fatty acid composition to queens but within the first week of hive life, they increase the polyunsaturate and decrease the monounsaturate lipid content of their membranes, possibly due to the activation of a genetic program and metabolic reprogrammation resulting from pollen consumption. This means that their membranes likely become more susceptible to lipid peroxidation in this first week of hive life. So, the results again support the suggestion that membrane composition might be an important factor in the determination of longevity. In another approach, these predictions have also been tested in a comparison among three wild type strains of *D. melanogaster* differing in their longevities (a long-lived strain: Oregon R, and two short-lived strains: Canton S and Dahomey). The results also confirm the presence of an inverse correlation between membrane unsaturation and lipoxidation-derived molecular damage and longevity. So, the greater the longevity of the *Drosophila* strain, the lower is the membrane unsaturation (Naudi et al., 2013).

Recent studies show that bivalves are excellent models for longevity research (Abele et al., 2009; Philipp and Abele, 2010). This taxonomic group includes the longest-living non-colonial metazoan (the Iceland clam *Arctica islandica*, MLSP = 507 years; Wanamaker et al., 2008), as well as surf clams (= Family Donacidae) with species of no more than 1 year longevity. Two traits make bivalves excellent models for longevity research: first, bivalves from temperate and cold-water environments can be accurately aged by counting their annual shell growth rings. This makes it possible to relate physiological state to chronological age in wild populations. Secondly, bivalve molluscs are genetically intermediate to classical invertebrate models of longevity (e.g., worms and flies) and mammals. This provides an opportunity to study the evolution of oxidative stress response pathways and animal longevity (Austad, 2009; Philipp and Abele, 2010). In this scenario, a recent study (Munro and Blier, 2012) analyzed the possible existence of a PI vs. longevity relationship by comparing the phospholipid fatty acid composition from mitochondrial membranes and other cell membranes of the longest-living metazoan species (*Arctica islandica*, 507 years) to four other sympatric bivalve molluscs also differing in their longevities (28, 37, 92, and 106 years, respectively). The results certified that long-lived marine bivalves possess peroxidation-resistant membranes. Indeed there is a significant inverse correlation between PI and longevity, analogously to the described findings from vertebrates (mammals and birds) (Hulbert et al., 2007).

A final specific comment concerning humans, as exceptionally long-lived species, is also pertinent. The findings from a recent work centered in offspring of long-lived individuals again seem to reinforce the association between membrane unsaturation and longevity. Thus, the fatty acid composition and PI of erythrocyte membranes from 41 nonagenarian offspring were compared with 30 matched controls (Puca et al., 2008). The results of this study demonstrated a lower PI in the lipid composition of erythrocyte membranes derived from nonagenarian offspring versus matched controls. This is indicative of reduced susceptibility to oxidative stress and increased membrane integrity at the cellular level for nonagenarian offspring compared with the general population under investigation. In this context, it should be plausible to infer that lipid composition of erythrocyte membranes could represent a useful biomarker of longevity. Finally, with the idea to investigate which specific lipids associate with familial longevity, another study (Gonzalez-Covarrubias et al., 2013) has explored the plasma lipidome in 1526 middle-aged offspring of nonagenarians (59 years) and 675 (59 years) controls from the Leiden Longevity Study. In men, no significant differences were observed between offspring and controls; whereas in women, 19 lipid species were related to familial longevity. More interestingly, the longevity-linked lipidomic profile showed by female offspring expressed a higher ratio of MUFA over PUFA lipid species revealing a plasma lipidome more resistant to oxidative stress.

Overall, all these comparisons (a) support an important role for membrane fatty acid composition in the determination of longevity, (b) reinforce the idea that the connection between membrane unsaturation and longevity is not restricted to vertebrates, and (c) suggest that membrane composition is regulated in a species-specific way.

### Are experimental extensions in longevity by genetic manipulations accompanied by attenuations of membrane unsaturation and lipoxidation-derived molecular damage?

The relevance of membrane unsaturation in determining longevity has also been recently reinforced by using Drosophila as an experimental model (Sanz et al., 2010). In this study, transgenic strains of Drosophila that express yeast NDI1ubiquitously were created (in yeast, the single-subunit NADH dehydrogenase Ndi1 serves as a non-proton-translocating alternative enzyme that replaces complex I, bringing about the reoxidation of intramitochondrial NADH). NDI1 expression bring about a decreased accumulation of lipoxidation-derived damage markers, accompanied by a reduced rate of ROS production and restored bioenergetic state, resulting in an increased longevity. This lower lipoxidation-derived damage in the long-lived strains is also linked to an adaptive response with a low degree in membrane unsaturation (Naudi et al., 2013).

In *C. elegans* as experimental model (Hulbert, 2011; Shmookler Reis et al., 2011), it was analyzed the fatty acid profile of lipids extracted from strains of *C. elegans* that vary in longevity by ~10-fold, and display several significant log-linear correlations between longevity and fatty acid composition. The results—strongly influenced by two mutant strains (daf-2 and age-1, both long-lived mutant strains linked to a dysruption of the insulin like-signaling pathway) that showed the greatest longevities—demonstrated that comparing the shortest-living with longest-living strains total MUFAs increased from 34 to 48%, total PUFAs decreased from 37 to 26%, and PI decreased from 141 to 81. All together, these findings suggest that mutations leading to higher longevities require of, at least, an adaptation of the degree of membrane unsaturation.

Functional assays, using RNAi to attenuate gene expression, might provide evidence that such genes (e.g., for desaturase or detoxifying enzymes) play causal roles in enhancing longevity. Longevity extensions permit stronger inferences than its decrease, due to the many ways longevity could be shortened upon disruption of any pathway that contributes to survival. In this context, two studies made on *C. elegans* related to the PUFA biosynthesis and detoxifiying enzymes for RCS deserve a special mention.

As mentioned above, RCS are compounds produced under oxidative stress conditions. These compounds are detoxified in multiple ways, including conjugation to glutathione, oxidation by aldehyde dehydrogenases, or reduction by aldoketoreductases (Pamplona, 2008). Reaction of RCS with gluthathione can proceed in one of two ways: by nonenzymatic conjugation or through glutathione transferase (GST)-mediated conjugation to form Michael adducts. GSTs belong to a supergene family of multifunctional enzymes, which are particularly involved in the detoxification of highly reactive aldehydes (Sheehan et al., 2001). Interestingly, protection against oxidative stress is the major driver of positive selection in mammalian GSTs, explaining the overall expansion pattern of this enzymes' family. The biological relevance of this protein-enzyme family is highlighted by studies in *C. elegans* demonstrating that interference with the expression of these enzymes significantly shortens the longevity of the organism and increases the formation of lipoxidation-derived protein adducts (Ayyadevara et al., 2007), whereas the overexpression of GSTs increases the longevity (Ayyadevara et al., 2005). Consequently, in this approach, the relationship between longevity and the expression of the detoxifiying enzymes indicates that the substrate of that enzyme, reactive carbonyl compounds, may be causally involved in limiting longevity. In the other approach, *C. elegans* benefits from RNAi suppression of genes encoding either of two elongases or a delta-5 desaturase, fat-4, whereas knochdowns of delta-9 desaturase genes can slightly reduce longevity (Shmookler Reis et al., 2011). Taken together, these functional data imply that the modulation of fatty acid composition to increase resistance to lipid peroxidation is one of the mechanisms for longevity extension.

### Are experimental extensions in longevity by nutritional interventions accompanied by attenuations of membrane unsaturation and lipoxidation-derived molecular damage?

This question is a key issue that goes beyond correlation to establish a causative role for membranes and lipoxidative stress in the determination of longevity. In order to clarify whether the low membrane unsaturation of long-lived animals protects their cellular components from lipid oxidation and lipoxidation-derived molecular damage, studies of experimental dietary modification of *in vivo* membrane fatty acid unsaturation have been performed (Herrero et al., 2001; Portero-Otin et al., 2003; Pamplona et al., 2004). These studies were specially designed to partially circumvent the homeostatic system of compensation of dietary-induced changes in membrane unsaturation which operates at tissue level. The obtained findings demonstrate that lowering the membrane unsaturation of cellular membranes protects tissues against lipid peroxidation and lipoxidation-derived macromolecular damage.

Available evidences in favor for a relationship between membrane unsaturation and longevity proceed from nutritional interventions that extend longevity in experimental models. So, caloric (CR), as well as protein (PR) and methionine (MetR) restriction attenuates age-related changes in the degree of membrane unsaturation and the level of lipoxidation products in a variety of tissues and animal species (Yu, 2005; Pamplona and Barja, 2006, 2011; Jové et al., 2013a,b). Thus, a decrease in membrane unsaturation, lipid peroxidation and lipoxidation-derived damage has been reported in tissues like liver, heart, and brain from these dietary restrictions in rats and mice (Laganiere and Yu, 1987; Pamplona et al., 2002a,b,c; Lambert et al., 2004; Sanz et al., 2005, 2006; Ayala et al., 2007; Gómez et al., 2007; Naudí et al., 2007; Caro et al., 2008, 2009; Jové et al., 2013a,b). CR has also been shown to reduce levels of lipofuscin in tissues of rodents and *C. elegans* (Enesco and Kruk, 1981; De et al., 1983; Rao et al., 1990; Terman and Brunk, 2004; Gerstbrein et al., 2005).

From these studies it can be inferred that the magnitude of the change is modest for membrane unsaturation (between 2.5–10%) compared to that for the lipoxidation-derived molecular damage (between 20–40%) likely due to the added effect of the lower mitochondrial ROS generation also induced by these nutritional interventions. In addition to the moderate but significant effect on membrane unsaturation, these nutritional interventions show an effect directly related to the intensity of the dietary restriction applied, being both PR and MetR even more intense and effective that CR. It is suggested from available data that the effects of CR on membrane unsaturation could be divided in three stages depending of CR duration in rats. During short-term CR periods, decreases in the rate of mitochondrial ROS production and lipoxidation-derived protein damage are observed in some tissues together with minor changes in membrane fatty acid composition. If CR is applied for several weeks-months, changes in particular fatty acids with moderate or no changes in PI occur, although the magnitude of the changes depends on the organ and the intensity of the restriction. Finally, in long-term CR, the beneficial effects on ROS production, PI and lipoxidation-derived damage are evident. In fact, CR diminishes the slope of the relationship between age and age-related lipid peroxidation. Thus, the CR manipulation seems to trigger an adaptive response protecting the most basic requirements of membrane integrity.

Very valuable information could be also obtained from studies designed to modify membrane fatty acid composition in order to increase membrane unsaturation and to evaluate its impact in animal longevity. Thus, in a recent study (Tsuduki et al., 2011) the influence of long-term ingestion of fish oil (a PUFAn-3 rich oil) on lipid oxidation and longevity was examined in senescence-accelerated (SAMP8) mice. Male mice were fed a fish oil diet (5% fish oil and 5% safflower oil) or a safflower oil diet (10% safflower oil) from 12 weeks of age. The SAMP8 mice fed fish oil showed a significantly reduced longevity, in association with a higher lipid peroxidation, when compared to mice fed safflower oil.

Reinforcing this outcome, there is also a reduction in *C. elegans* longevity with the addition of PUFAs to their diet (Shmookler Reis et al., 2011). Thus, adult worms were maintained on agar plates spotted with *E. coli* (strain OP50). Control plates were unsupplemented, while treatment plates contained either palmitic acid (16:0) or eicosapentaenoic acid (20:5*n*-3). The results showed that longevity in the presence of 20:5*n*-3 was reduced 20% relative to unaugmented controls, and by 16% relative to worms supplemented with 16:0. In an independent experiment, supplementation with 22:6*n*-3 produced similar reductions in survival, 24% relative to untreated controls and 15% relative to palmitic acid. In contrast to these results, in another study (Hillyard and German, 2009) it is described a beneficial effect of these molecules (20:5*n*-3 and 22:6*n*-3) when added to the short-lived mutant *fat-3*, which lacks a functional delta-6 desaturase, and thus PUFAs including 20:5*n*-3.

## Mechanism responsible for the longevity-related differences in membrane unsaturation

The low PI observed in long-lived species are due to changes in the type of unsaturated fatty acid that participates in membrane composition (Pamplona et al., 2002a,b,c; Hulbert et al., 2007). Globally, there is a systematic redistribution between the types of PUFAs present from highly unsaturated fatty acids such as 22:6*n*-3, 20:5*n*-3, and 20:4*n*-6 in short-lived animals to the less unsaturated 18:3*n*-3, 18:2*n*-6, and 18:1 in the long-lived ones, at mitochondrial and tissue level. Furthermore, the PI of the respective diets did not correlate with longevity. This indicates again that the contribution of the variations in the degree of unsaturation of dietary fats to the inter-species differences is, if any, very modest.

The mechanisms responsible for the longevity-related differences in fatty acid profile can be related, in principle, to the PUFAs biosynthesis pathway (including desaturases, elongases, and peroxisomal beta-oxidation; see Figure 1), and the deacylation-reacylation cycle. The available estimates of delta-5 and delta-6 desaturase activities, as well as different elongase activities, indicate that they are several folds lower in long-lived species than in short-lived ones (Pamplona et al., 2002a,b,c; Pamplona and Barja, 2003; Hulbert et al., 2007; Shmookler Reis et al., 2011). This can explain why e.g., 22:6*n*-3 and 20:4*n*-6 decreases and 18:2*n*-6 and 18:3*n*-3 increases, from short-to long-lived animals, since elongases/desaturases are the rate-limiting enzymes of the *n*-3 and *n*-6 pathways synthesizing the highly unsaturated PUFAs 20:4*n*-6 and 22:6*n*-3 from their dietary precursors, 18:2*n*-6 and 18:3*n*-3, respectively. Thus, elongation-desaturation pathways would make available *in situ* the *n*-6 and *n*-3 fatty acids to phospholipid acyltransferases in order to remodel the phospholipid acyl groups. In addition, a relevant role for peroxisomal beta-oxidation, a metabolic pathway key for the obtention of 20:5*n*-6 and 22:6*n*-3 should be also considered to the light of recent results.

In accordance with this interpretation, a recent study (Jobson et al., 2010) with a phylogenomic approach to identify the genetic targets of natural selection for extended longevity in mammals has been published. The premise of this work is that genes preventing high rates of aging should be under stronger selective pressure in long-lived species, relative to short-lived ones. In other words, long-lived species should allow identifying a stronger level of amino acid conservation from specific genes than short-lived ones. The results obtained by comparing the nonsynonymous and synonymous evolution of 5.7 million codon sites across 25 species proved that genes involved in fatty acid biosynthesis (elongases, desaturases, and lipoxidation repair), as well as extracellular collagen composition, have collectively undergone increased selective pressure in long-lived species, whereas genes involved in DNA replication/repair or antioxidants, among others, have not.

More investigations are, however, needed to confirm the role of these metabolic pathways in aging and longevity, and to extend them to the potential role of regulatory and transcriptional factors involved in PUFA metabolism.

## The biological membrane as dynamic structural adaptive system

**Figure 3:**
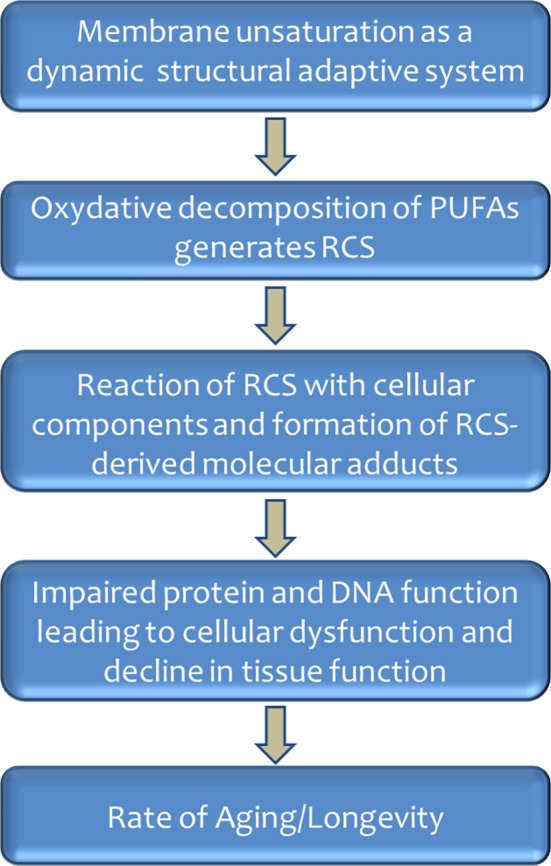
**Role of membrane unsaturation and lipoxidation-derived molecular damage in aging and longevity**.

Animals with a high longevity have a low degree of membrane fatty acid unsaturation based on the redistribution between types of PUFAs. This may be viewed as an elegant evolutionary strategy, because it decreases the sensitivity to lipid peroxidation and lipoxidation-derived damage to cellular macromolecules without strongly altering fluidity/microviscosity, a fundamental property of cellular membranes for the proper function of e.g., receptors, ion pumps, and transport of metabolites. This would occur because membrane fluidity increases acutely with the introduction of the first and less with the second double bond (due to their introduction of “kinks” in the fatty acid molecule), whereas additional (the third and following) double bonds cause few further variations in fluidity (Brenner, 1984). This is so because the kink has a larger impact on fluidity when the double bond is situated near the center of the fatty acid chain (first double bond) than when it is situated progressively nearer to its extremes (next double bond additions). In the case of the sensitivity to lipid peroxidation, however, PI increase irrespective of the double bond location at the center or laterally on the fatty acids (Holman, 1954). Thus, by switching fatty acids with four or six double bonds by those having only one, two (or even three) double bonds, the sensitivity to lipid peroxidation is strongly decreased in long-lived animals, whereas the fluidity of the membrane would be essentially maintained. This hypothesis, reminiscent of membrane acclimation to different environments at PUFA level in poikilotherms and bacteria, has been termed *homeoviscous longevity adaptation* (Pamplona et al., 2002a,b,c), and displays the biological membranes as a dynamic structural adaptive system (see Figure 3).

### Conflict of interest statement

The authors declare that the research was conducted in the absence of any commercial or financial relationships that could be construed as a potential conflict of interest.
